# Synthesis, Structure, and Thermoelectric Properties of Quaternary Selenides M_2_In_5_Sb_9_Se_23_ (M = Sn, Pb) with NaCl-Type Ribbon Motifs

**DOI:** 10.1021/acs.inorgchem.5c05769

**Published:** 2026-05-14

**Authors:** Yen-Han Huang, Guan-Ruei Chen, Chi-Shen Lee

**Affiliations:** † Department of Applied Chemistry, College of Science, 34914 National Yang Ming Chiao Tung University, Hsinchu 300093, Taiwan; ‡ Materials Science Group, 57815 National Synchrotron Radiation Research Center, Hsinchu 300092, Taiwan

## Abstract

Two quaternary selenides, M_2_In_5_Sb_9_Se_23_ (M = Sn, Pb), were synthesized by solid-state routes and characterized by single-crystal and synchrotron powder X-ray diffraction. Both compounds crystallize in orthorhombic *Pbam* (No. 55) and comprise alternating NaCl-(111) ribbons of edge-sharing (In/Sb)­Se_6_ octahedra and NaCl-(100) ribbons of distorted (Pb/Sn/Sb)–Se polyhedra that pack in a herringbone-like structure feature. Diffuse-reflectance spectroscopy indicates a narrow optical gap of 0.91 eV for Pb_2_In_5_Sb_9_Se_23_. Transport measurements on cold-pressed, unoriented Pb_2_In_5_Sb_9_Se_23_ indicate p-type semiconducting behavior with an Arrhenius activation energy of *E*_a_ ∼ 0.25 eV, Seebeck coefficients up to +460 μV K^–1^ (≈440 K), and intrinsically low total thermal conductivity of ∼0.5 W m^–1^ K^–1^, yielding *zT* ∼ 0.005 at 440 K. Density functional calculations for both analogues predict an indirect band gaps and ribbon-localized band-edge states, suggesting anisotropic electronic transport. These results identify mixed NaCl-(111)/(100) ribbon assembly as a motif in multinary selenides and establish M_2_In_5_Sb_9_Se_23_ as a useful platform for structure–property analysis and future thermoelectric optimization.

## Graphical abstract

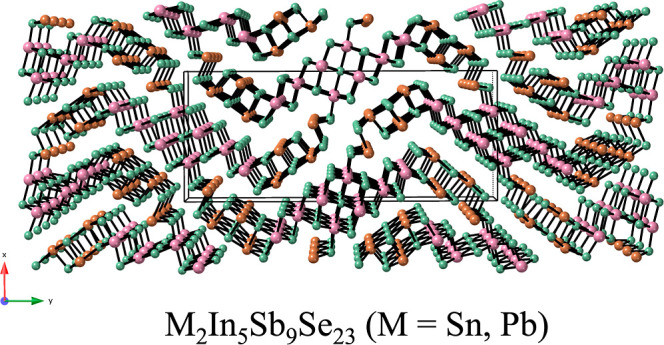

## 1 Introduction

Multinary chalcogenides, comprising three or more elements combined with chalcogen atoms (S, Se, Te), have long attracted attention due to their structural complexity and wide range of physical properties.^1–4^ Their diversity originates from variations in cation size, electronegativity, and oxidation state, which dictate coordination environments and bonding interactions.^5–7^ This structural and compositional flexibility provides a fertile platform for the discovery of new functional materials, particularly for applications in energy conversion and information technologies.^8,9^ Chalcogenide compounds already underpin several technological applications. In photovoltaics, CdTe and CuInSe_2_ are very useful solar-cell materials, while Cu_2_ZnSn­(S,Se)_4_ has emerged as a sustainable alternative composed of earth-abundant elements.^10–13^ In electrocatalysis, layered MoS_2_ offers edge-site activity with near-ideal hydrogen adsorption energies, and CoSe_2_ exhibits bifunctional reactivity toward both hydrogen and oxygen evolution.^14,15^ Thermoelectric chalcogenides such as SnSe,^16^ with record figures of merit (*zT* > 2), exemplify the potential of this class of materials; performance enhancements in multinary chalcogenide AgPb_18_SbTe_20_,^17^ Cu_2_Se,^18^ and Pb_7_Bi_4_Se_13_ ^19^ have been achieved through defect engineering and liquid-like ion transport. In nonlinear optics, compounds such as HgGaSe_2_ and GaSe enable frequency conversion in the mid-infrared region.^20,21^ In addition, phase-change materials including Ge_2_Sb_2_Te_5_, Sb_2_Te_3_, and GeTe underpin nonvolatile memory devices by exploiting rapid and reversible structural transitions.^22,23^

Our previous work has centered on the synthesis and characterization of multinary selenides within homologous series constructed from NaCl-type structural fragments.^24–29^ Compounds such as Sn_2_Pb_5_Bi_4_Se_13_ and Sn_8.65_Pb_0.35_Bi_4_Se_15_ were identified as members of the lillianite series, while Pb_4_In_*x*_M_6–*x*_Se_13_ (M = Bi, Sb) and A_4_B_10_Se_19_ (A = Sn, Pb; B = Sb, Bi) expanded the cannizzarite family.^26,30^ More recently, we have reported homologous series incorporating step-layered building units, including A_10_B_18_Se_37_ (A = Sn/Pb; B = In/Sb/Bi) and M_2_Sb_5_Bi_5_Se_17_ (M = Sn, Pb), highlighting the versatility of NaCl-type stacking strategies in constructing complex multinary selenides.^25,27,31–33^ Despite these advances, quaternary selenides that integrate group 13 (In), group 14 (Sn/Pb), and group 15 (Sb/Bi) elements remain relatively underexplored, with only a few reported examples such as InSn_2_Bi_3_Se_8_, In_0.2_Bi_1.8_Sn_6_Se_9_, and Pb_4_In_2_Sb_4_Se_13_.^30,34^

In this study, we extend the chemistry of multinary chalcogenides to the synthesis of new quaternary selenides, M_2_In_5_Sb_9_Se_23_ (M = Sn, Pb). These compounds crystallize in the orthorhombic space group *Pbam* (No. 55) and feature distinctive NaCl-type ribbon motifs interconnected into anisotropic frameworks. Despite many NaCl-fragment chalcogenides, compounds that coassemble NaCl-(111) and NaCl-(100) ribbons into a single 3D framework remain scarce. Their structural complexity, incorporation of heavy elements, and narrow-gap semiconducting character suggest promising prospects for thermoelectric optimization.

## 2 Experimental Section

### 2.1 Synthesis

Elemental Sn/Pb, In, Sb, and Se were combined in stoichiometric ratios under an inert atmosphere and sealed in evacuated fused-silica tubes (5–10 Pa). The mixtures were heated from room temperature to 873 K over 6 h, held for 48 h, and furnace-cooled to ambient temperature. The products were then reground, resealed under vacuum, and annealed at 823 K for an additional 24 h. Single crystals of Pb_2_In_5_Sb_9_Se_23_ were obtained under these conditions. Attempts to obtain phase-pure bulk Sn_2_In_5_Sb_9_Se_23_ by varying reaction time/temperature or using alternative growth approaches yielded mixtures of binary/ternary selenides; therefore, bulk property measurements for the Sn analogue were not performed due to insufficient phase purity.

### 2.2 Single-Crystal X-ray Diffraction

Single-crystal X-ray diffraction (SCXRD) data were collected for selected crystals of Sn_2_In_5_Sb_9_Se_23_ and Pb_2_In_5_Sb_9_Se_23_. Structures were solved by direct methods and refined by full-matrix least-squares refinement on *F* ^2^. Crystallographic parameters are summarized in Table 1 and atomic coordinates/interatomic distances are provided in Tables S1 and S2. The crystallographic data have been deposited at the Cambridge Crystallographic Data Centre under CCDC 2514592 (Sn_2_In_5_Sb_9_Se_23_) and 2514598 (Pb_2_In_5_Sb_9_Se_23_). These data can be obtained free of charge from the Cambridge Crystallographic Data Centre at http://www.ccdc.cam.ac.uk/data_request/cif.

**Table 1:** Crystal Data and Conditions of Data-Collection Conditions for M_2_In_5_Sb_9_Se_23_ (M = Sn, Pb)

chemical formula	Sn_2_In_5_Sb_9_Se_23_	Pb_2_In_5_Sb_9_Se_23_
formula weight	3723.31	3914.58
temperature	296(2) K	
wavelength	0.71073 Å	
crystal size	0.02 × 0.05 × 0.09 mm^3^	0.02 × 0.05 × 0.10 mm^3^
crystal system	orthorhombic	
space group, *Z*	*Pbam* (55), 2	
unit cell dimensions	*a* = 14.586(8) Å	*a* = 14.594(2) Å
	*b* = 34.973(12) Å	*b* = 35.039(5) Å
	*c* = 3.996(2) Å	*c* = 4.0158(4) Å
measuring θ_min,max_/deg	1.16/25.03°	2.23/26.37°
volume	2038.4(17) Å^3^	2053.5(5) Å^3^
density (calculated)	6.066 g/cm^3^	6.331 g/cm^3^
absorption coefficient	30.365 mm^–1^	37.582 mm^–1^
max. and min transmission	0.5819 and 0.0290	0.5203 and 0.0235
*F* (000)	3172	3310
index ranges	–17 ≤ *h* ≤ 16	–18 ≤ *h* ≤ 18
	–40 ≤ *k* ≤ 41	–43 ≤ *k* ≤ 43
	–4 ≤ *l* ≤ 4	–2 ≤ *l* ≤ 5
theta range for data collection	1.16°–25.03°	2.23°–26.37°
reflections collected	9878	18255
independent reflections	2082 [*R*(int) = 0.0616]	2389 [*R*(int) = 0.0866]
goodness-of-fit on *F* ^2^	1.129	1.053
final *R* indices [*I* > 2σ(*I*)]	*R*1 = 0.0446 w*R*2 = 0.1178	*R*1 = 0.0379 w*R*2 = 0.0626
*R* indices (all data)	*R*1 = 0.0710 w*R*2 = 0.1563	*R*1 = 0.0644 w*R*2 = 0.0699
largest diff peak/hole/e^–^ Å^–3^	2.580/–2.241	3.631/–3.645

### 2.3 Characterization

Phase identification was performed by laboratory PXRD and synchrotron PXRD. Rietveld refinements were carried out using TOPAS with the Thompson–Cox–Hastings pseudo-Voigt peak-shape function.^35–38^ Preferred orientation was modeled using the March–Dollase approach. Morphology and texture were examined by SEM, and semiquantitative compositions of selected crystals were checked by SEM–EDS to support phase assignment (Figure S2). Bulk elemental ratios for Pb_2_In_5_Sb_9_Se_23_ were additionally assessed by ICP–AES, and the results are consistent with the nominal composition within experimental uncertainty (Table S3). Thermal stability was evaluated by DTA using sealed quartz capsules. Samples were heated to 1173 at 10 K min^–1^ and cooled to 573 at 10 K min^–1^ under flowing N_2_.

### 2.4 Physical Properties

All physical property measurements reported herein (diffuse reflectance, electrical conductivity, Seebeck coefficient, and thermal transport) were performed on Pb_2_In_5_Sb_9_Se_23_ pellets; bulk property measurements for the Sn analogue were not obtained due to phase-purity limitations. Diffuse-reflectance spectra were converted to the Kubelka–Munk function *F*(*R*) and analyzed using Tauc plots to extract the optical band gap *E*_g_ from the absorption edge. DC electrical conductivity (σ) was measured from 300 to 600 K using a four-point probe configuration. The temperature uncertainty in the measurement region was ≤1 K. Seebeck coefficients (*S*) were measured from 300–600 K under dynamic vacuum. Transport specimens were prepared as cold-pressed, unoriented polycrystalline pellets (cuboids, ∼1 × 2 × 5 mm^3^) pressed under a 1-ton load, achieving >80% of the theoretical density. No hot pressing or spark plasma sintering (SPS) was used; therefore, residual porosity and grain-boundary resistance may suppress σ and the derived power factor and *zT* relative to fully dense polycrystalline material. Thermal diffusivity (α) was measured on cold-pressed round pellets (12.50 mm diameter, ∼0.80 mm thickness), and total thermal conductivity was calculated as κ_total_ = αρ*c*_p_, where ρ is the measured density and *c*_p_ was obtained from DSC. The electronic contribution was estimated via the Wiedemann–Franz relation κ_e_ = *L*σ*T*. The thermoelectric figure of merit was calculated as *zT* = *S* ^2^σκ_total_^–1^T.^39^

### 2.5 Electronic Structure Calculations

Electronic structure calculations were performed for M_2_In_5_Sb_9_Se_23_ (M = Sn, Pb) using DFT as implemented in WIEN2k (LAPW + lo).^40–44^ Exchange–correlation effects were treated within the PBE–GGA. To construct charge-balanced models suitable for electronic-structure analysis, mixed-occupancy sites were represented by ordered configurations, and the calculations were performed in a reduced-symmetry cell (*P*2/*m*) to treat the mixed sites as fully occupied crystallographic positions. Band structures and densities of states were computed using appropriately dense *k*-point sampling.^45,46^ Spin–orbit coupling (SOC) was not included in these calculations. Bonding analysis was conducted via COHP using the Stuttgart TB–LMTO–ASA method.^46–49^

## 3 Results and Discussion

### 3.1 Synthesis and Structural Characterization

Single-crystal X-ray diffraction (SCXRD) analysis of selected needle-like crystals successfully identified a new quaternary chalcogenide with a nearly charge-balanced composition close to Pb_2_In_5_Sb_9_Se_23_. The refinements indicate substantial mixed-site occupancies of Pb/Sb and In/Sb across all metal sites. Guided by this structural result, we attempted to prepare a pure phase of the idealized composition Pb_2_In_5_Sb_9_Se_23_, which was reproducibly obtained under the sealed-tube synthesis conditions used here (Figure S1). Subsequent experiments aimed at replacing Pb with Sn yielded single crystals of a structurally analogous phase, confirming the existence of the Sn-containing compound. However, phase-pure bulk Sn_2_In_5_Sb_9_Se_23_ could not be isolated under the conditions explored, and the products consistently contained binary and/or ternary selenide impurities (Table S4). Consequently, the bulk transport measurements presented below are limited to the Pb analogue, whereas the Sn analogue is discussed on the basis of crystallographic analysis and electronic-structure calculations.

To examine the compositional tolerance of this structure type, additional reactions were carried out with varied In/Sb ratios in the M–In–Sb–Se quaternary system. Powder X-ray diffraction indicated that Sb-rich starting compositions more readily produced the M_2_In_5_Sb_9_Se_23_-type phase, whereas more In-rich compositions favored the formation of Sb-substituted β-In2Se3 and related secondary phases. Despite systematic attempts, no clear evidence was obtained for an extended solid solution of generalized formula M_2_(In/Sb)_14_Se_23_. These results represent limited composition screening under the sealed-tube conditions and indicate that Pb_2_In_5_Sb_9_Se_23_ is the composition most reproducibly obtained as the dominant bulk phase under the present synthesis conditions.

Comparable site-occupancy patterns were obtained for the two analogues, and the mixed Sn/Sb and In/Sb assignments in the Sn-containing phase were modeled in analogy to the Pb analogue. The present crystallographic analysis establishes the crystal structure and stoichiometric model, whereas the precise distribution of In/Sn/Sb across individual mixed sites remains less certain without complementary element-sensitive structural probes, as these elements have very similar X-ray scattering factors.

The structural analysis of the synchrotron PXRD data was performed using the Rietveld refinement method. Initial refinement was based on the atomic positions determined from single-crystal XRD data of Pb_2_In_5_Sb_9_Se_23_. The refinement yielded a good fit to the observed data, with a weighted-profile *R*-factor (*R*_wp_) of 2.074% (Figure 1). Nevertheless, several weak residual peaks (with intensities less than 5% of the strongest reflection) remained unindexed. These unassigned features likely originate from minor secondary phases or stacking faults, reflecting the inherent complexity and sensitivity of multinary selenide systems to slight compositional or synthetic variations. Scanning electron microscopy (SEM) imaging revealed that the polycrystalline samples primarily consisted of rod-shaped crystallites with sharply defined edges, indicative of anisotropic crystal growth. In addition, energy-dispersive X-ray spectroscopy (EDS) elemental mapping demonstrated homogeneous distributions of Pb, In, Sb, and Se throughout the samples. The uniformity observed in EDS analyses supports the chemical homogeneity and phase integrity of the synthesized material (Figure S2 and Table S3).

**Figure 1:**
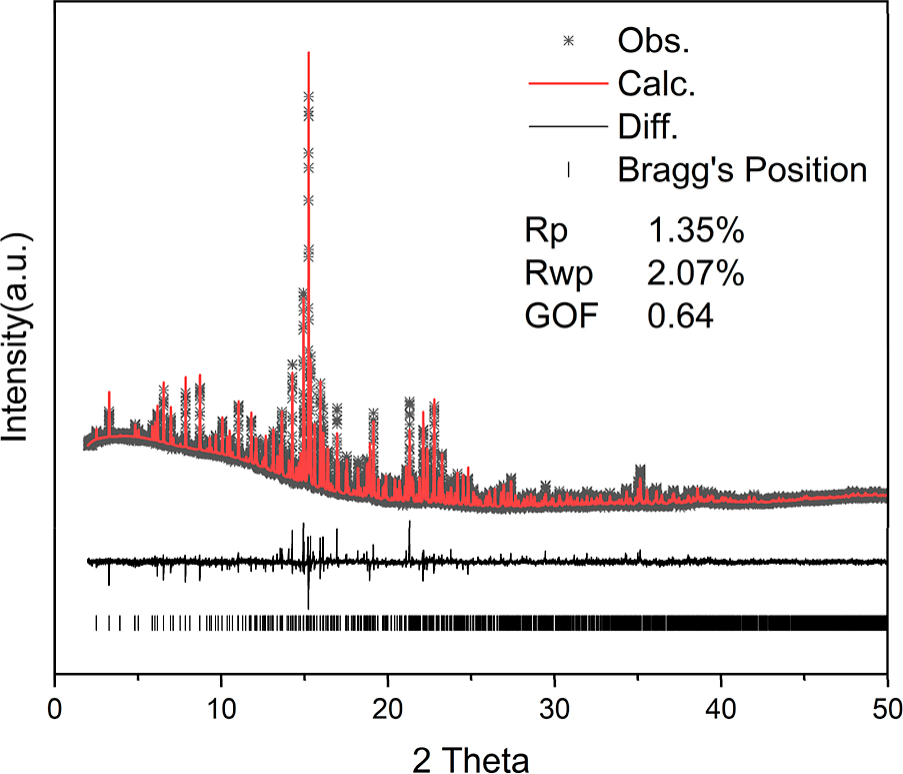
Rietveld refinement of synchrotron XRD pattern of Pb_2_In_5_Sb_9_Se_23_. Black circles represent the observed data, and the red line represents the calculated profile. Inset: SEM image of Pb_2_In_5_Sb_9_Se_23_.

**Figure 2:**
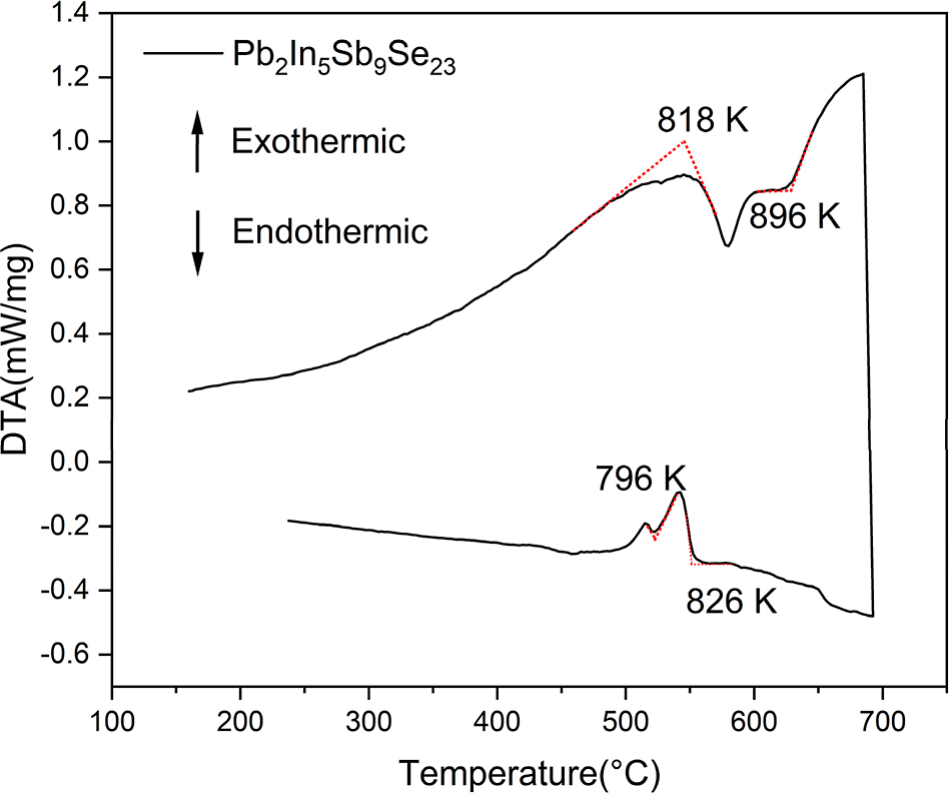
DTA heating and cooling curves of Pb_2_In_5_Sb_9_Se_23_.

Differential thermal analysis (DTA) was utilized to evaluate the thermal stability of Pb_2_In_5_Sb_9_Se_23_ (Figure 2). DTA reveals a first endothermic event beginning at 818 K, followed by a second event near 896 K, indicating that the phase is not stable at higher temperatures under these measurement conditions. During cooling, an exothermic peak appeared between 796 and 826 K, reflecting partial recrystallization from the decomposed material. Post-DTA powder X-ray diffraction confirmed that the sample no longer remains as a single phase after this thermal cycle and contains secondary decomposition product(s). These results indicate that Pb_2_In_5_Sb_9_Se_23_ is accessible within the sealed-tube synthesis and annealing temperature for the synthesis of a pure phase, but its high-temperature stability is limited. The present data define a practical upper thermal boundary below the DTA onset for sustained operation (Figure S3).

X-ray photoelectron spectroscopy (XPS) analysis was performed to examine the oxidation states of the constituent elements in Pb_2_In_5_Sb_9_Se_23_ (Figures 3 and S4). The measured binding energies of Pb 4f (137.9 and 142.9 eV), In 3d (444.36 and 451.96 eV), Sb 3d (529.6 and 539.0 eV), and Se 3p/3d (165.7, 160.4, 55.0, and 54.1 eV) are consistent with Pb^2+^, In^3+^, Sb^3+^, and Se^2–^ oxidation states, respectively.^27,50–55^ The absence of satellite peaks or significant peak shifts suggests no secondary phases or unexpected valence states. These results are consistent with the proposed charge-balanced formula and chemical homogeneity of the synthesized crystals, in agreement with the structural model derived from X-ray diffraction.

**Figure 3:**
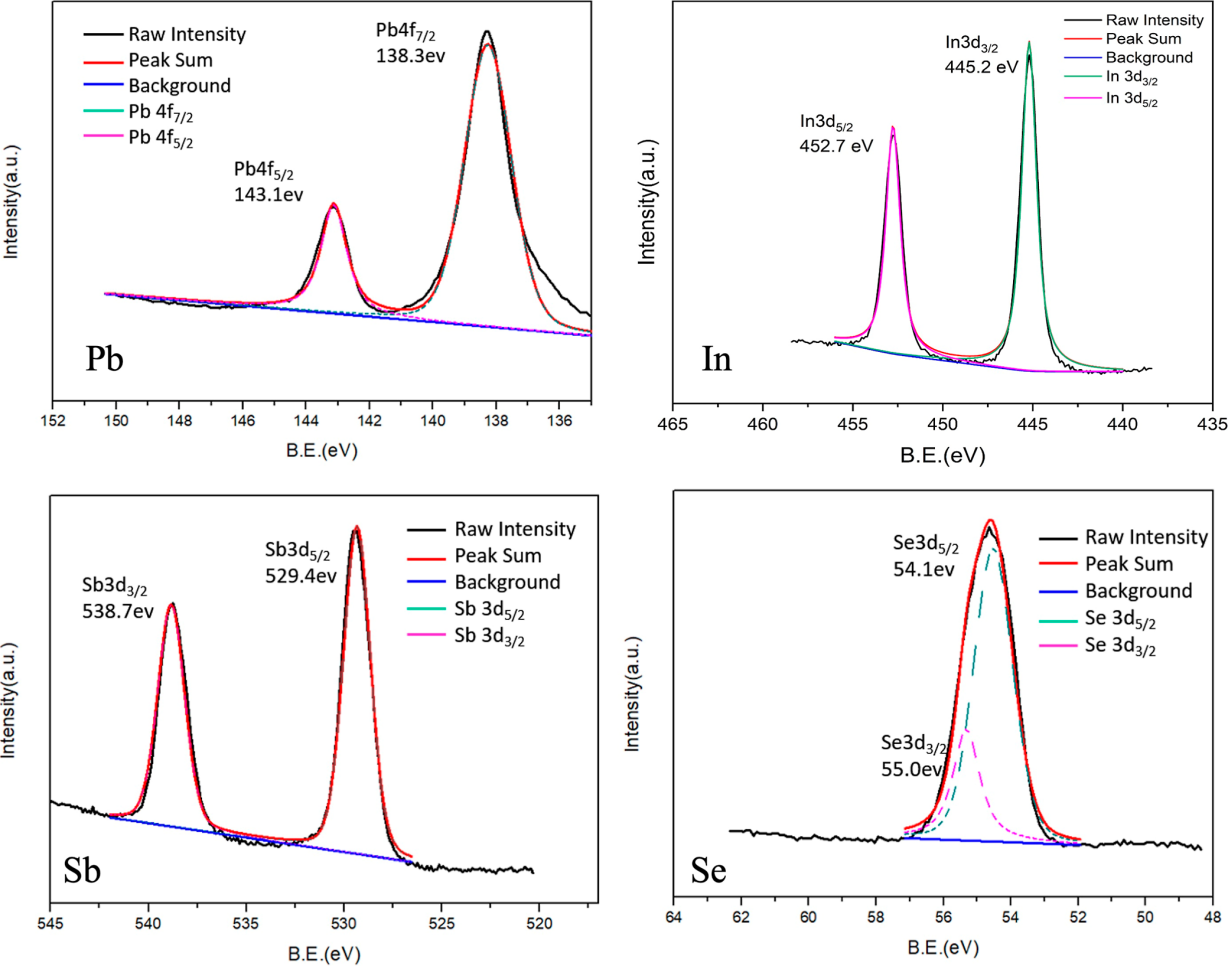
X-ray photoelectron spectra of Pb_2_In_5_Sb_9_Se_23_.

### 3.2 Structure Description

The crystal structure of M_2_In_5_Sb_9_Se_23_ (M = Sn, Pb) represents a novel class of complex multinary selenides (Figure 4). These compounds crystallize in the orthorhombic system (space group *Pbam*, No. 55) and contain 20 crystallographically distinct sites: eight cation positions—four mixed In/Sb sites (M1–M4) and four mixed (Pb/Sn)/Sb sites (M5–M8)—and 12 selenium sites. Using Pb_2_In_5_Sb_9_Se_23_ as an illustrative example, most of which occupy 4a Wyckoff positions, except for Se1 located at a 2a Wyckoff site. The framework is constructed from two 1D modules: a NaCl-(111) type ribbon of edge-sharing (In/Sb)­Se_6_ octahedra and a NaCl-(100) type ribbon comprising distorted (Pb/Sb)–Se polyhedra. These ribbons alternate along b and pack in a herringbone-like arrangement, yielding the stoichiometric combination [M_8_Se_6_Se_2/2_]­[M_8_Se_15_Se_2/2_] → M_16_Se_23_ per repeat. The (Pb/Sb) polyhedra exhibit pronounced off-centering, consistent with lone-pair activity of ns^2^ cations (Pb^2+^, Sb^3+^). The unit cell is highly anisotropic (Table 1), with the *c* axis shorter than *a* and *b*, consistent with a ribbon-based framework. Within the NaCl-(111)/(100) ribbon fragments, short M–Se contacts are predominantly <3 Å, whereas inter-ribbon separations are longer (typically ∼ 3.4–3.9 Å), implying stronger electronic coupling within/along ribbons than across them. However, the present transport measurements were performed on unoriented polycrystalline pellets and thus provide an orientation-averaged response.

**Figure 4:**
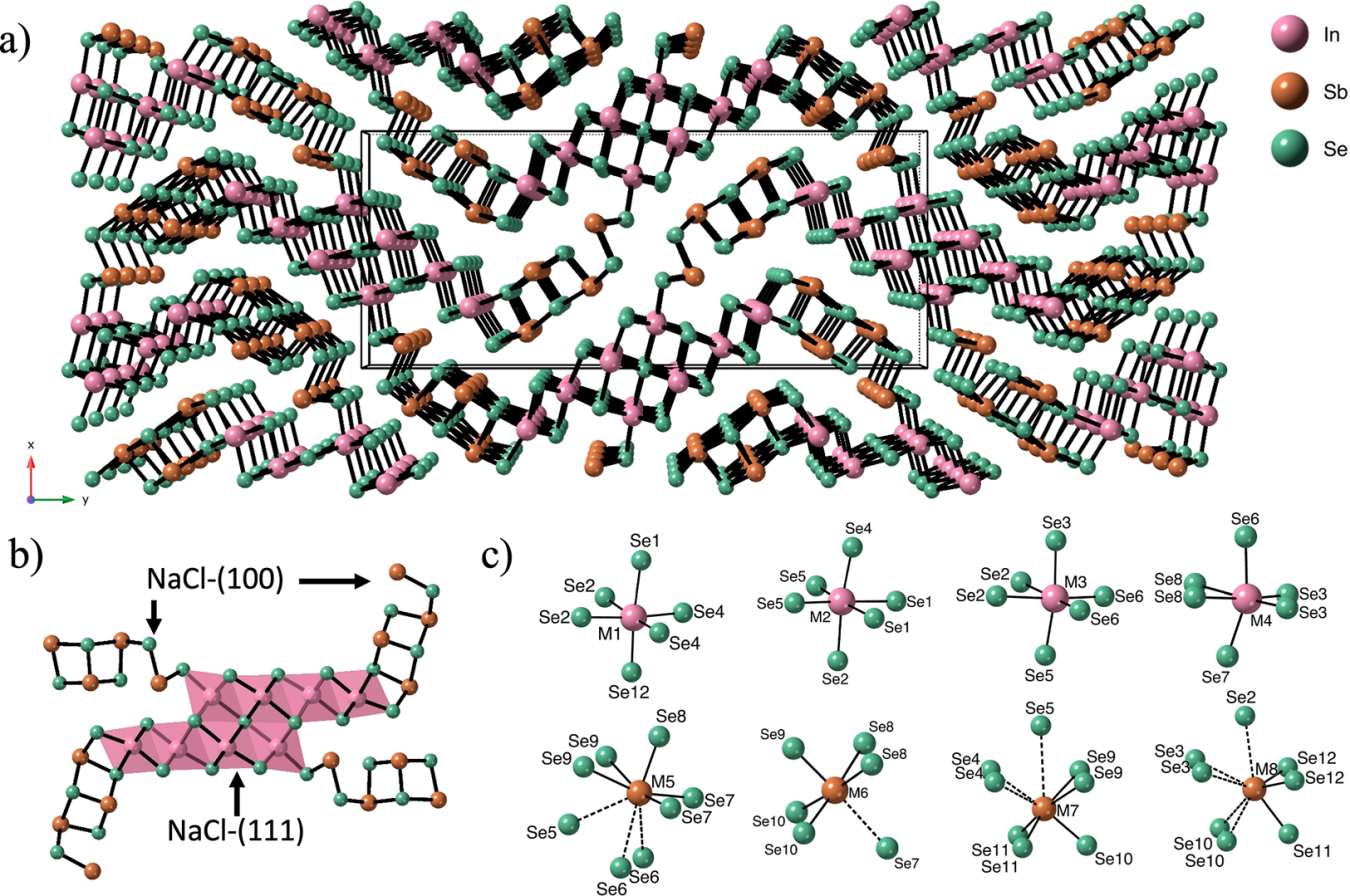
Crystal structure of Pb_2_In_5_Sb_9_Se_23_: (a) view along the *c* axis, (b) building units of the puckered layered structure, and (c) representative coordination environments.

The local environments around the metal centers exhibit considerable diversity. The M1–M4 sites, occupied by In and Sb, adopt distorted octahedral geometries with M–Se bond lengths ranging from 2.59 to 3.08 Å. The M5–M8 positions (Pb/Sb) show a range of polyhedral configurations, including trigonal pyramidal, square pyramidal, and distorted antiprismatic shapes. M5, M7, and M8 exhibit elongated antiprismatic coordination with M–Se distances up to ∼3.16 Å, while M6 adopts a square pyramidal geometry featuring five short bonds (2.74–2.90 Å) and one longer bond (∼3.07 Å). These variations arise from the stereochemical activity of lone pairs on Pb^2+^ and Sb^3+^, inducing local off-centering and bonding anisotropy. All selenium sites are fully occupied and play crucial roles in bridging the polyhedral units. Among them, Se7, Se8, and Se12 exhibit highly asymmetric environments and serve as pivotal connectors between the ribbons, contributing to structural distortion and enhancing vibrational complexity.

Calculations of bond valence sum (BVS) were carried out to help the assignment of metal sites for M_2_In_5_Sb_9_Se_23_ (M = Sn, Pb).^56,57^ The BVS values of M1–M4 sites were 2.5–3.8 for In/Sb, and 2.0–3.60 for Sn/Pb, which were deviated from the expected +2 for Sn/Pb and +3 for In/Sb observed in binary phases of SnSe/PbSe and In_2_Se_3_/Sb_2_Se_3_.^58–61^ The BVS values of Se sites ranged from 1.7 to 2.1 for the Se 2- parameters. Because all metal sites exhibit mixed occupancies (In/Sb on NaCl-(111) ribbons; Pb/Sn/Sb on NaCl-(100) ribbons), bond-valence sums deviate from formal valences and are provided in Table S5 for completeness; they should not be overinterpreted for site assignment in this covalent/mixed-occupancy lattice.^62,63^ These results showed poor BVS agreement with a model with mixed occupied metal sites.

The NaCl-(111) type ribbons, formulated as [M_8_Se_15_Se_2/2_] (M = In, Sb), consist of edge-sharing distorted octahedra aligned along the ribbon axis. Due to similar scattering factors, In and Sb occupy these sites in an approximately 1:1 ratio. Their bond distances fall within typical ranges and resemble those found in Bi_2_Se_3_ and M_4_In_5_Sb_9_Se_25_.^28^ In contrast, the NaCl-(100) type ribbons, represented by [M_8_Se_6_Se_2/2_] (M = Pb, Sb), feature square pyramidal and trigonal pyramidal units, all of which are occupied by mixed Pb/Sb atoms. Structural distortions in these motifs are also attributed to lone pair effects, consistent with behaviors seen in PbSe and Sb_2_Se_3_. Each ribbon comprises two atomic layers, contributing to the material’s low-dimensional structural characteristics. These two types of ribbons alternate along the crystallographic *b*-axis, forming a herringbone-like superstructure. The resulting three-dimensional network is zigzagging and corrugated, constructed through shared Se atoms that link adjacent ribbons. This arrangement reflects the staggered layer alignment commonly seen in complex chalcogenides.^64–71^

Overall, the structure can be expressed with the general formula [M_8_Se_6_Se_2/2_]­[M_8_Se_15_Se_2/2_], identifying M_2_In_5_Sb_9_Se_23_ as a new member in a homologous series of multinary selenides. Single-crystal XRD confirms this structural model, with refinement results consistent with the ideal stoichiometry. Notably, the crystals exhibit a preferred orientation along the [001] direction, indicating significant anisotropy during growth—a factor that may impact directional transport properties. Compared to other multinary selenides, M_2_In_5_Sb_9_Se_23_ uniquely integrates NaCl-(100) and NaCl-(111) type structural motifs within a unified three-dimensional scaffold. While similar zigzag patterns have been observed in compounds such as InSbS_3_,^72^ and Pb_5_In_8_Se_17_,^73,74^ they typically rely solely on NaCl-(111) type units. Conversely, Pb_2_Sb_2_Se_5_ is dominated by NaCl-(100) type arrangements.^75^

Building units of alternative NaCl-(111) and NaCl-(100) type structures have been identified in multinary chalcogenides with various arrangements. The stripes of NaCl-(111) type units are overlapped by steps to accommodate the variation in the overlapping of octahedral polyhedra, which are filled with various sizes of NaCl-(100) type units to form a three-dimensional framework. Our previous studies with a step-layered structure exhibiting two isolated octahedra include Pb_2_Sb_5_Bi_5_Se_17_,^27^ Sn_4_In_5_Sb_9_Se_25_, and Sn_6.13_Pb_1.87_In_5.00_Sb_10.12_Bi_2.88_Se_35_.^28^ The concurrent presence of both building blocks in M_2_In_5_Sb_9_Se_23_ represents a notable new structural feature that extends the architectural complexity of multinary chalcogenides (Figure S5).

### 3.3 Electronic Structure

Electronic structures of both analogues (Sn and Pb) were obtained by DFT using a charge-balanced structural model, providing a comparative framework for the experimental transport measurements, which were performed only on the Pb analogue. Figure 5 shows the calculated electronic band structure, the corresponding total/partial density of states (DOS/PDOS), and the crystal orbital Hamilton population (COHP) for Pb_2_In_5_Sb_9_Se_23_, where the dashed line marks the Fermi energy (*E*_F_). The results reveal a narrow indirect band gap of 0.43 eV, with the conduction-band minimum located at the *U* point and the valence-band maximum occurring between Γ and *U*. This band alignment indicates semiconducting behavior, consistent with experimental expectations for related selenide systems. The DFT-calculated gap is smaller than the optical gap of 0.91 eV obtained from diffuse reflectance. This difference is expected, as the DFT value is based on an idealized model and does not consider temperature, disorder, or excitonic effects, which can underestimate band gaps. Accordingly, the calculations are used here primarily to identify the indirect-gap topology and the ribbon-dominated character of the near-edge states. The Sn analogue, Sn_2_In_5_Sb_9_Se_23_, likewise exhibits an indirect band gap (Figure S7a).

**Figure 5:**
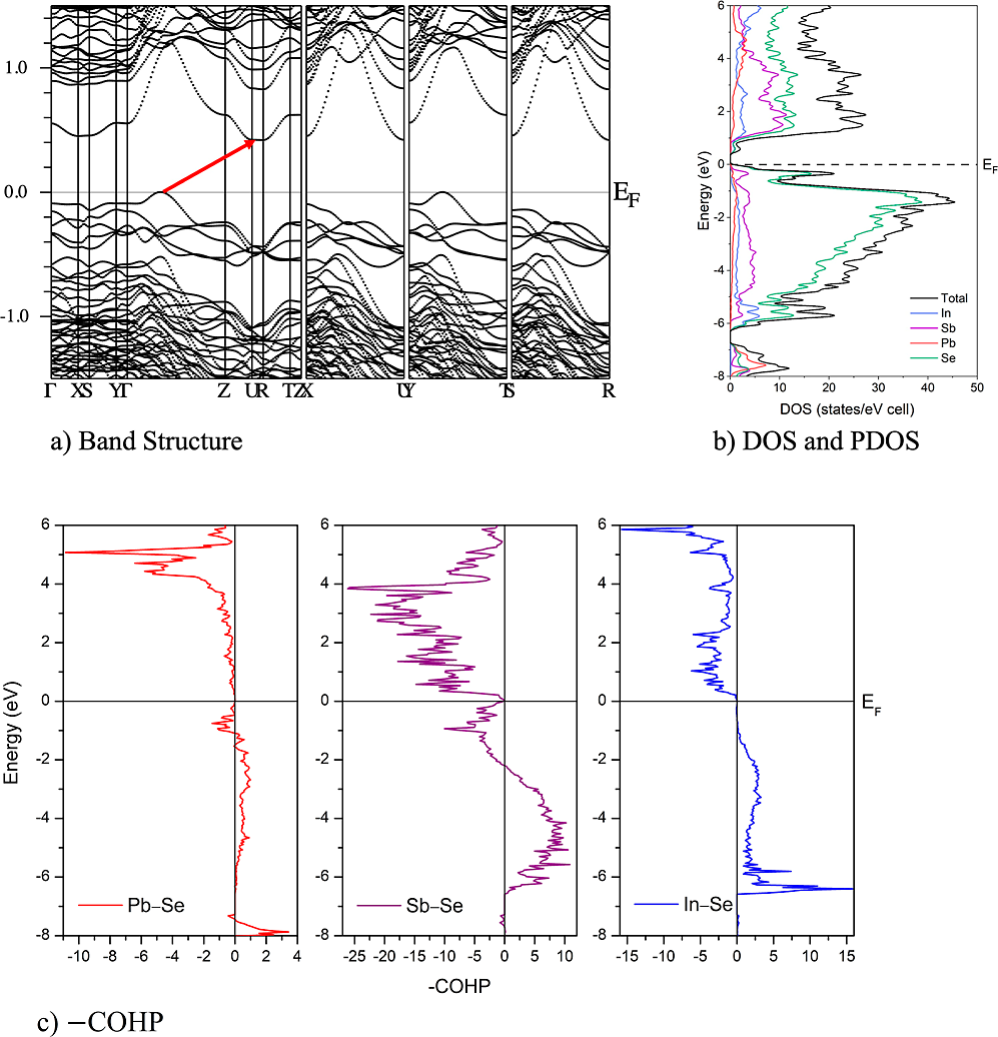
Electronic-structure results for Pb_2_In_5_Sb_9_Se_23_: (a) band structure, (b) total and partial density of states, and (c) crystal orbital Hamilton population. The dashed line indicates the Fermi energy (*E*_F_).

The near-VBM region is relevant for transport in the p-type case. Along the high-symmetry path, the valence-band edge comprises several closely spaced maxima. For Pb_2_In_5_Sb_9_Se_23_ (Figure 5), at least two near-VBM maxima lie within approximately 0.0–0.2 eV of the highest valence states, whereas the Sn analogue shows a more converged near-VBM manifold with multiple maxima of comparable energy (Figure S7a). Such near-degeneracy may affect hole degeneracy and enhance the DOS near the VBM, which is favorable for Seebeck coefficients at a given hole concentration, provided that mobility is not overly suppressed.

The DOS/PDOS further clarifies the orbital character at the band edge. In Pb_2_In_5_Sb_9_Se_23_ (Figure 5b), the valence band (approximately −6.5 to 0 eV) is dominated by hybridized Se p and Sb p contributions, with smaller In and Pb contributions near the band edge. Bonding interactions are evident in the PDOS, particularly between metal (Pb/In/Sb) p orbitals and Se 4p orbitals in the −5.0 to −1.0 eV energy window. A comparatively narrow feature immediately below the VBM is consistent with localized ns^2^ lone-pair character (notably associated with Pb/Sb), which contributes weakly to Se hybridization and is commonly linked to electronic asymmetry and enhanced lattice anharmonicity in heavy post-transition-metal chalcogenides. The Pb/Sb COHP (Figure 5c) also confirms antibonding states near the Fermi level, which could be conducive to the p-type behavior. Meanwhile, the In COHP exhibits bonding states below the Fermi level, favoring the formation of the structure. Above the Fermi level, the conduction bands are dominated by the unoccupied p orbitals of Pb, In, and Sb, with a minor contribution from Se p states near the conduction-band minimum. Such ribbon-dominated band-edge character, together with the anisotropic bonding metrics, suggests that intrinsic carrier transport may be direction-dependent. The corresponding DOS/PDOS for Sn_2_In_5_Sb_9_Se_23_ is provided in Figure S7b and shows a similar band-edge orbital makeup, while the more converged VBM manifold in Figure S7a suggests a higher effective p-type degeneracy along the sampled path. The COHP for Sn_2_In_5_Sb_9_Se_23_ in Figure S7c reveals a similar appearance compared to Pb_2_In_5_Sb_9_Se_23_, except for more bonding states on the Sn site, implying higher stability at the bonding level. However, the stress arising from differences in atomic size and the presence of other metastable phases eventually render the purification of Sn_2_In_5_Sb_9_Se_23_ unfeasible.

### 3.4 Physical Properties

Given these synthesis outcomes, we restricted transport measurements to Pb_2_In_5_Sb_9_Se_23_.

UV–vis diffuse reflectance spectroscopy was employed to probe the optical absorption properties of powdered Pb_2_In_5_Sb_9_Se_23_ (Figure 6). The experimentally measured reflectance data were transformed into a pseudoabsorption coefficient using the Kubelka–Munk (K–M) function. Extrapolation of the fitted linear segment yields an indirect optical band gap of approximately 0.91 eV, with a high coefficient of determination (*R* ^2^ = 0.997).^30,76,77^ In contrast, the corresponding direct-transition representation exhibits inferior linearity and a larger apparent gap energy, indicating that a direct transition does not adequately describe the absorption behavior of Pb_2_In_5_Sb_9_Se_23_. The experimentally determined optical gap is qualitatively consistent with the DFT results, as both indicate indirect-gap semiconducting behavior. The band gap derived from diffuse reflectance corresponds to the optical absorption onset and can be sensitive to the fitting window and the intrinsic difference between optically allowed transitions and the fundamental electronic gap.

**Figure 6:**
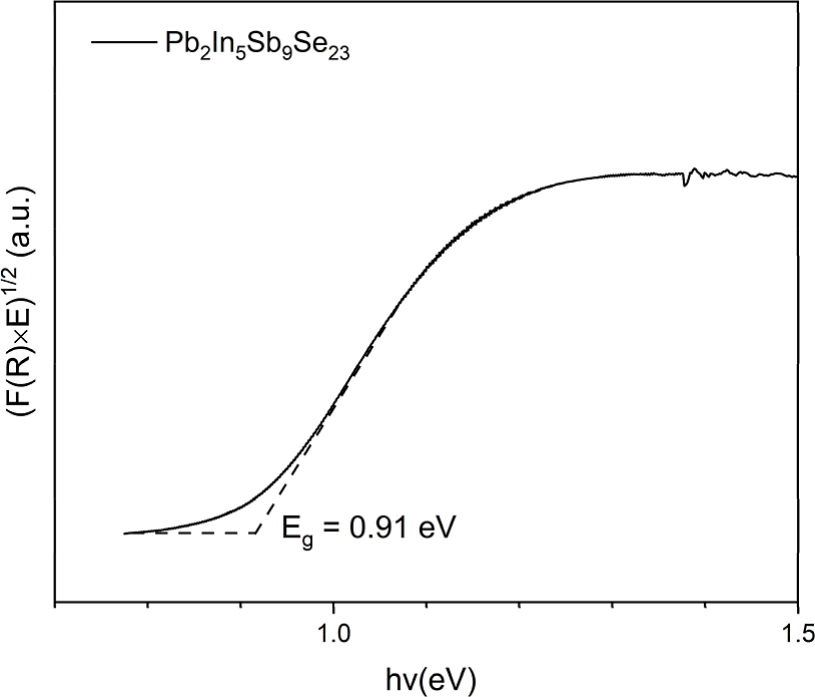
Diffuse-reflectance UV–vis/near-IR spectrum of Pb_2_In_5_Sb_9_Se_23_.

The thermoelectric properties of Pb_2_In_5_Sb_9_Se_23_ were evaluated by measuring the electrical conductivity (σ), Seebeck coefficient (*S*), thermal diffusivity (α), and thermal conductivity (κ), from which the power factor (PF) and dimensionless figure of merit (*zT*) were derived (Figure 7). The electrical conductivity increases steadily between 300 and 600 K, displaying thermally activated behavior consistent with semiconducting transport and an Arrhenius activation energy of *E*_a_ ∼ 0.25 eV. The calculated activation energy is derived from cold-pressed polycrystalline pellets, which may be influenced by defect/disorder states and grain-boundary barriers. The Seebeck coefficient remains positive throughout the investigated range, increasing from 280 μV K^–1^ at 300 K to 460 μV K^–1^ at 440 K, thereby confirming p-type conduction. Hall-effect measurements were not available in the present study; therefore, the carrier concentration is discussed only as an order-of-magnitude estimate based on the equation σ = *ne*μ, yielding a low carrier concentration of *n* ∼ 10^16^–10^17^ cm^–3^ at 300 K. Although σ is modest compared with conventional thermoelectrics, the maximum power factor of ∼0.02 μW·cm^–1^·K^–2^ is observed at 410 K (Figure S6), which is substantially lower than structurally related layered/homologous low-κ chalcogenides that retain PF values by sustaining much higher electrical conductivities.^19,78–82^ The measured total thermal conductivity, κ_total,_ is consistently low, with values near 0.5 W·m^–1^·K^–1^ across the investigated temperature range. The electronic contribution estimated from κ_e_ = *L*σ*T* is negligible because of the low electrical conductivity. Accordingly, thermal conductivity is predominantly phononic in origin, and κ_total_ = κ_L_ + κ_e_ ∼ κ_L_ within experimental uncertainty.^83^ The combination of low κ and moderate electronic transport produces a maximum *zT* of ∼0.005 at 440 K. Because the transport data were obtained from cold-pressed, unoriented pellets with relative densities of only about 80% of theoretical value, the measured electrical conductivity, power factor, and *zT* should be regarded as lower-bound polycrystalline values. Residual porosity and grain-boundary resistance likely suppress charge transport in the present specimens, and the reported transport performance should therefore be interpreted as a baseline for this framework rather than an optimized thermoelectric result. For comparison, Bi_2_Te_3_–Sb_2_Te_3_ alloys (*zT* = 1.2–1.4 near 373 K),^84–86^ CsBi_4_Te_6_ (*zT* = 0.8 near 320 K),^87^ AgPb_*m*_SbTe_2+*m*_ nanocomposites (*zT* ≈ 2.2 at 800 K),^17^ and p-type SnSe^16^ single crystals (*zT* = 2.6 at 923 K) represent benchmark systems. The lower performance of Pb_2_In_5_Sb_9_Se_23_ is primarily limited by electrical conductivity, which could be improved by optimizing carrier concentration, microstructure, or single-crystal growth. Although the resulting *zT* values remain modest, the combination of low thermal conductivity and semiconducting transport identifies the ribbon-based Pb_2_In_5_Sb_9_Se_23_ framework as a chemically informative platform for structure–property analysis, while further improvements in densification, carrier concentration control, and directional transport measurements will be required to evaluate its intrinsic thermoelectric potential.

**Figure 7:**
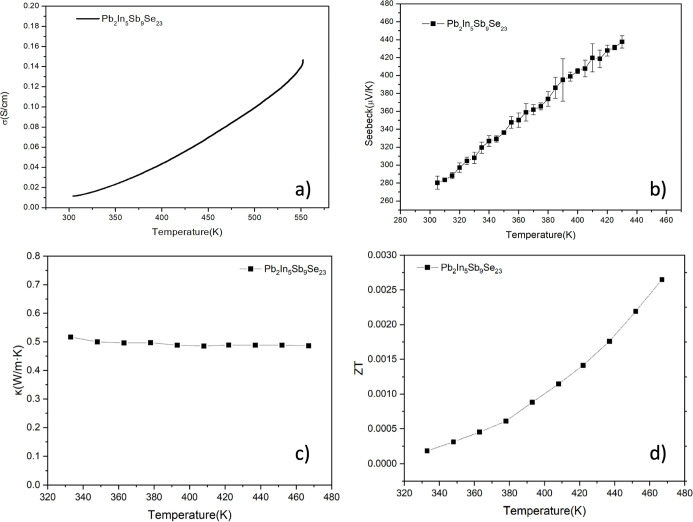
Thermoelectric properties of Pb_2_In_5_Sb_9_Se_23_: (a) electrical conductivity, (b) Seebeck coefficient, (c) thermal conductivity, and (d) dimensionless figure of merit (*zT*).

## 4 Conclusion

We have synthesized and structurally characterized a new multinary selenide, M_2_In_5_Sb_9_Se_23_ (M = Sn, Pb), which crystallizes in the orthorhombic space group *Pbam* (No. 55) and features a distinctive architecture composed of alternating NaCl-type ribbon units. The structure integrates NaCl-(111) type edge-sharing octahedra filled with In^3+^/Sb^3+^ and NaCl-(100) type motifs hosting Pb^2+^/Sn^2+^ and Sb^3+^ in distorted polyhedra, interconnected via shared Se atoms into an anisotropic, herringbone-like framework. This modular, compositionally tunable structure promotes phonon scattering via heavy elements, coordination diversity, and lone-pair-induced distortions, thereby reducing lattice thermal conductivity. Transport measurements on the Pb analogue, together with DFT for both analogues, confirm semiconducting behavior. While Pb_2_In_5_Sb_9_Se_23_ exhibits p-type thermopower and intrinsically low κ, Sn_2_In_5_Sb_9_Se_23_ is presently supported by structural determination and calculated band structure. The reported transport values are based on cold-pressed pellets; better densification is expected to enhance σ, the power factor, and *zT*. Although the current power factors are moderate, these measurements serve as a benchmark for the intrinsic effects of the ribbon-based framework (structural complexity, mixed occupancy, and lone-pair distortions) on electronic transport and lattice thermal conductivity. This study exemplifies a structurally rich and flexible platform for designing advanced multinary chalcogenides, offering valuable insights into the development of thermoelectric materials and broader energy-related applications.

## Supplementary Material



## Notes

(1) Experimental and simulated PXRD patterns, (2) total and partial DOS, (3) atomic coordinates, (4) interatomic distances (PDF)
