# Supplementary material for: Synthesis, Structure, and Thermoelectric Properties of Quaternary Selenides M2In5Sb9Se23 (M = Sn, Pb) with NaCl-Type Ribbon Motifs
Source: Inorg Chem. 2026 May 14;65(20):10914–24. doi: 10.1021/acs.inorgchem.5c05769 (PMC13213906; doi:10.1021/acs.inorgchem.5c05769)
Supplement: Supplementary file 1 [file ic5c05769_si_001.pdf]

Supporting Information

Synthesis, Structure, and Thermoelectric Properties of Quaternary Selenides $M_2In_5Sb_9Se_{23}$ (M = Sn, Pb) with NaCl-Type Ribbon Motifs

Yen-Han Huang[a,†], Guan-Ruei Chen[b,†], and Chi-Shen Lee[a]*

[a]Department of Applied Chemistry, College of Science, National Yang Ming Chiao Tung University, Hsinchu 300093, Taiwan

[b]Materials Science Group, National Synchrotron Radiation Research Center, Hsinchu 300092, Taiwan

* Corresponding author: chishen@nycu.edu.tw

E-mail address: chishen@nycu.edu.tw

ORCID: https://orcid.org/0000-0002-8848-5203

† Yen-Han Huang and Guan-Ruei Chen contributed equally to this work.

Experimental Section

General Synthesis Procedures

All synthesis operations were conducted in a glove box maintained under a dry nitrogen atmosphere, rigorously excluding oxygen and moisture. High-purity reagents, including Pb/Sn (99.9%, Alfa Aesar), In (99.9%, Alfa Aesar), Sb (99.9%, Alfa Aesar), and Se (99.9%, Alfa Aesar), were utilized without further purification. The initial synthetic strategy was based on exploratory reactions in the Pb–In–Sb–Se system, motivated by structural analogies with $Pb_3In_xSb_{14-x}Se_{24}$ (x = 5, 6). Stoichiometric mixtures of the elements were homogenized by mortar and pestle, loaded into fused-silica tubes, evacuated to 5-10 Pa, and flame-sealed. The samples (total batch ~0.5 g) were heated from room temperature to 873 K over 6 h, held for 48 h, furnace-cooled, reground, resealed, and annealed at 823 K for an additional 24 h. Under these conditions, single crystals of $Pb_2In_5Sb_9Se_{23}$ were reproducibly obtained. Additional reactions were then carried out to probe the compositional and synthetic window of the new phase. For the Sn analogue, variations in reaction time, annealing temperatures, and related growth conditions were examined; although single crystals of the Sn-containing phase were obtained, phase-pure bulk $Sn_2In_5Sb_9Se_{23}$ was not obtained , and the products remained mixtures containing binary and ternary selenides. The corresponding screening results are summarized in **Table S4**.

Single-Crystal X-ray Diffraction

Single-crystal X-ray diffraction data were collected using a Bruker APEX II CCD diffractometer equipped with graphite-monochromated Mo Kα radiation (λ = 0.71073 Å). Needle-shaped crystals were mounted for analysis. Data collection involved exposure durations of 10 s/frame, a crystal-to-detector distance of 6.0 cm, and θ range of 2.33° to 26.37°. Data completeness

exceeded 98% using Phi and Omega scans. Structure solutions and refinements were performed using SHELXS direct methods and full-matrix least-squares refinement on F^2 in APEX2.[1] Absorption corrections were applied using empirical surface-fitting, with atomic positions determined by differential Fourier synthesis.[2] Structural symmetry and quality were confirmed via APEX2.

Characterizations

Laboratory PXRD patterns were collected on a Bruker D8 Advance diffractometer using Cu Kα radiation (λ = 1.5418 Å; 40 kV, 40 mA) over 2θ = 10–80° with a step size of 0.025° and a counting time of 1.3 s per step. Synchrotron PXRD was measured at NSRRC BL01C2 using X-rays with λ = 0.6888 Å (18 keV). Powders were loaded into 0.3 mm diameter capillaries and data were collected with a Mar345 image plate detector. LaB_6 was used as an instrumental standard for calibration/correction. Rietveld refinements of the synchrotron data were performed using the SCXRD-derived model as the starting structure. Because the powder specimens exhibited pronounced rod-like morphology in SEM images, a March-Dollase preferred-orientation correction was included in the refinement. This correction improved the profile fit, but weak residual reflections remained, indicating that minor impurity phases and/or stacking-related disorder cannot be excluded completely.

SEM imaging was performed using JEOL JSM-7401F and Hitachi SU-8010 instruments. Semiquantitative elemental analyses of selected crystals were conducted using an EDS-equipped Hitachi S-4300 SEM. Bulk elemental ratios were measured by inductively coupled plasma atomic emission spectroscopy (ICP-AES) using an Agilent 725 spectrometer. Sample preparation and analytical details are provided alongside the ICP results table (**Table S4**). DTA

measurements were carried out using a Netzsch STA 409 PC Luxx system. Samples were sealed in quartz capsules placed in Al_2O_3 crucibles. Measurements were performed under flowing N_2, heating to 1173 K at 10 K min^{-1} and cooling to 573 K at 10 K min^{-1}.

Physical Properties

All physical property measurements reported herein (optical, electrical, Seebeck, and thermal transport) were performed on $Pb_2In_5Sb_9Se_{23}$ pellets. Bulk property measurements for the Sn analogue were not obtained due to limitations in phase purity. A SolidSpec-3700 DUV spectrophotometer with a diffuse reflectance accessory was used to measure the spectrum of the compound $Pb_2In_5Sb_9Se_{23}$ over the range 200-2000 nm at 298 K (25°C). The absorption data were calculated from the reflectance using the Kubelka–Munk function $F(R) = \alpha/S = (1-R)^2/2R$, where R is the reflectance at a given energy, a is the absorption, and S is the scattering coefficient. Diffuse-reflectance data were converted to an absorption proxy and analyzed using the Tauc formalism. The optical band gap E_g was obtained by linear fitting of the absorption-edge region in plots of $(F(R)\times E)^2$ (direct) or $(F(R)\times E)^{1/2}$ (indirect) versus photon energy $E(hv)$.

DC electrical conductivity (σ) analyses were conducted on $Pb_2In_5Sb_9Se_{23}$ using the standard four-point probe method, employing a homemade device over a temperature range of 300-600K (27 to 327 °C). The temperature uncertainty is within 1 K. Bulk samples were securely placed on a silica stage using a stainless steel-ceramic insulating sheet. These samples were then placed in a 4 cm-diameter silica chamber. Electrical contacts were established using four platinum wires that were affixed to the bulk samples with platinum glue. The samples underwent a vacuum treatment at 393 K for 10 minutes to ensure the platinum glue dried completely, enhancing contact performance. Once these preliminary steps were completed, the system was evacuated to $\sim 10^{-1}$

mbar and subsequently heated in a tubular furnace. Measurements were conducted with a Jiehan ECW-5600 electrochemical analyzer, which served as both the power source and voltage receiver. The current was set to 0.5 mA for the first measurement and 1.0 mA for the second. Hall-effect measurements were not performed; the carrier concentration n at 300 K was estimated from the measured electrical conductivity according to $\sigma = ne\mu$, where e is the elementary charge and μ is the carrier mobility. The mobility range of $\mu = 0.1\text{-}1$ cm^2 V^{-1}s^{-1} was adopted to represent the lower end expected for cold-pressed, moderately porous polycrystalline chalcogenide pellets.[3-5]

Seebeck coefficients (S) of $Pb_2In_5Sb_9Se_{23}$ were measured using a commercial MMR Technologies SB-100 system from 300 to 600 K under dynamic vacuum (~2 × 10^{-2} mbar). Constantan was used as an internal standard, and silver conductive paint was used to establish electrical contact. Transport specimens were prepared as cold-pressed, unoriented polycrystalline pellets (cuboids, ~1 × 2 × 5 mm^3) pressed under a 1-ton load, achieving relative densities >80% of the theoretical value. No hot pressing or spark plasma sintering (SPS) was used to densify the transport specimens; therefore, residual porosity and grain-boundary resistance may reduce the measured σ, power factor, and zT relative to fully dense polycrystalline material. Thermal diffusivity (α) of $Pb_2In_5Sb_9Se_{23}$ was measured with a Discovery Xenon Flash DXF 200 diffusivity analyzer (TA Instruments). Round pellet samples (12.50 mm diameter, approximately 0.80 mm thick) were cold pressed under a 5-ton force and coated with graphite paint. Thermal conductivity (κ_{total}) was calculated using $\kappa_{total} = \alpha\rho c_p$, with specific heat capacity (c_p) determined from DSC experiments and density (ρ) measured directly. The electronic thermal conductivity κ_e was estimated using the Wiedemann–Franz relation, $\kappa_e = L\sigma T$, where σ is the measured electrical conductivity and L is the Lorenz number. L was estimated from the measured Seebeck coefficient using a semiconductor-appropriate Seebeck-based approximation.[6] sing $\sigma \sim 0.01$ Scm^{-1} at 300 K

and L ~ 1.6×10^{-8} WΩK^{-2}, we obtain κ_e(300 K) ~ 4.8×10^{-6} W m^{1}K^{-1}, corresponding to < 0.001% of κ_{total}. The thermoelectric figure of merit (*zT*) was subsequently calculated using $zT = S^2\sigma\kappa^{-1}T$.[7]

Electronic Structure Calculations

Electronic structure calculations for $M_2In_5Sb_9Se_{23}$ (M = Sn, Pb) were carried out using density functional theory (DFT) as implemented in the WIEN2k package with the LAPW+lo basis set. [8-10] Initial self-consistent-field calculations were performed within the Perdew-Burke-Ernzerhof generalized gradient approximation (PBE-GGA) for exchange and correlation. [11]Because the experimental structure (space group *Pbam*) contains mixed-occupancy metal sites that cannot be treated directly in a fully ordered DFT model, charge-balanced ordered structural models were constructed from the experimental structure in a reduced-symmetry *P2/m* cell, in which the mixed sites were represented as fully occupied crystallographic positions. After convergence of the PBE calculations, the modified Becke-Johnson (mBJ) exchange potential was applied to refine the electronic structure and obtain improved band-gap estimates.[12] Initial self-consistent field (SCF) calculations used a 50-k-mesh, with structural optimizations performed using the MSR1a method. Convergence thresholds were set at 0.0001 Ry (energy), 1 mRy (force), and 0.001 e (charge). Band structures and density-of-states (DOS) calculations were performed using denser k-point grids and following high-symmetry k-paths.[13, 14] Spin-orbit coupling (SOC) was not included. Accordingly, the calculated electronic structures are used primarily for qualitative interpretation of the indirect-gap topology and band-edge orbital character. [15] COHP analyses were performed using the Stuttgart TB–LMTO–ASA program (version 4.7), which utilizes the tight-binding (TB) linear muffin-tin orbital (LMTO) method within the atomic-sphere approximation (ASA). The von

Barth–Hedin local density approximation (LDA) was employed for the exchange-correlation potential.[14, 16-18]

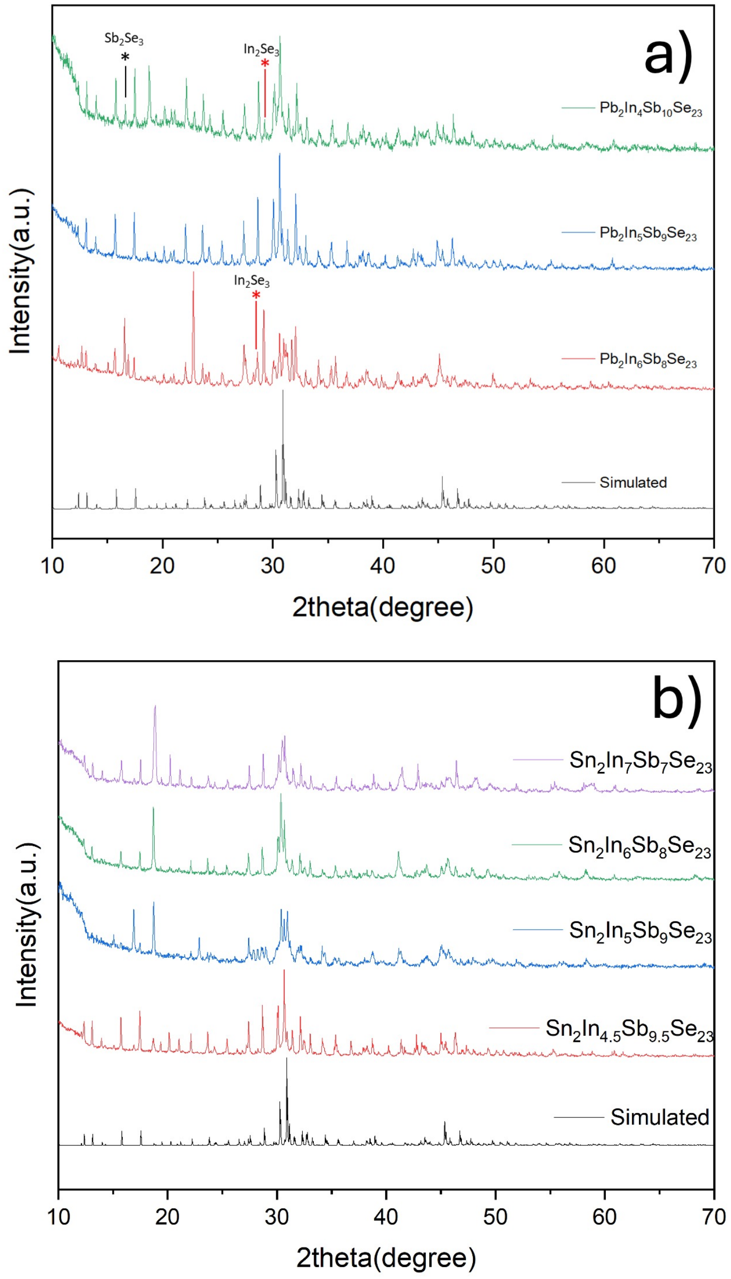

Figure S1. Comparison of experimental and simulated powder X-ray diffraction patterns for a) $Pb_2In_xSb_{15-x}Se_{23}$ (x = 4, 5, 6) and b) $Sn_2In_xSb_{15-x}Se_{23}$ (x = 4.5, 5, 6, 7).

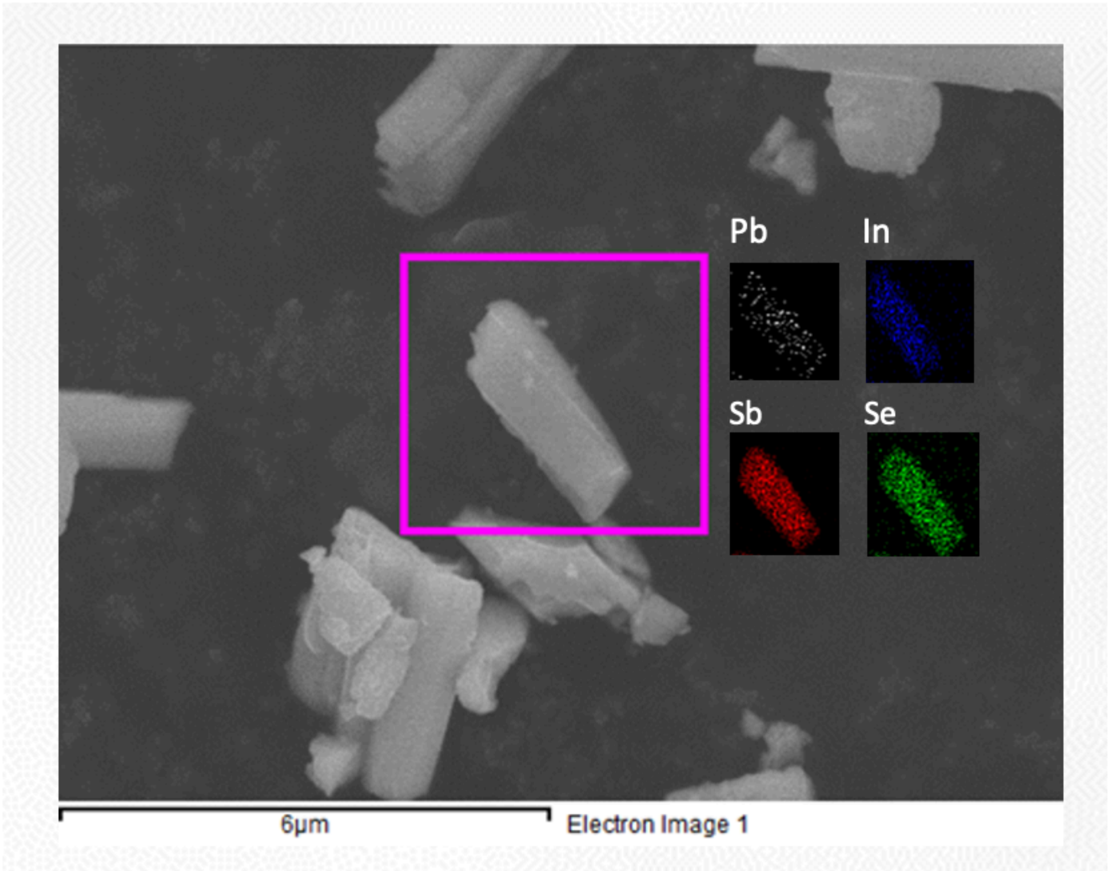

Figure S2 SEM image and EDX elemental maps of $Pb_2In_5Sb_9Se_{23}$.

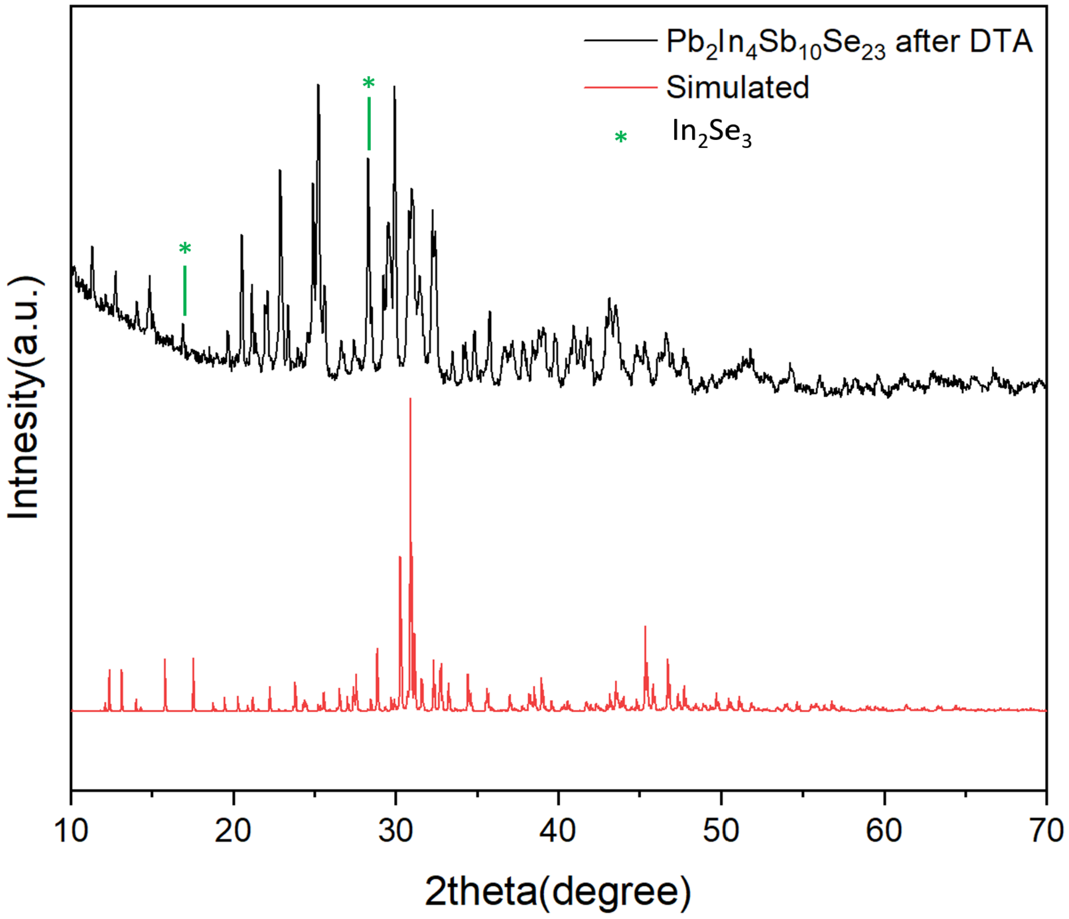

Figure S3. Powder X-ray diffraction pattern of $Pb_2In_5Sb_9Se_{23}$ after DTA measurement, showing reflections from the main phase and the impurity phase.

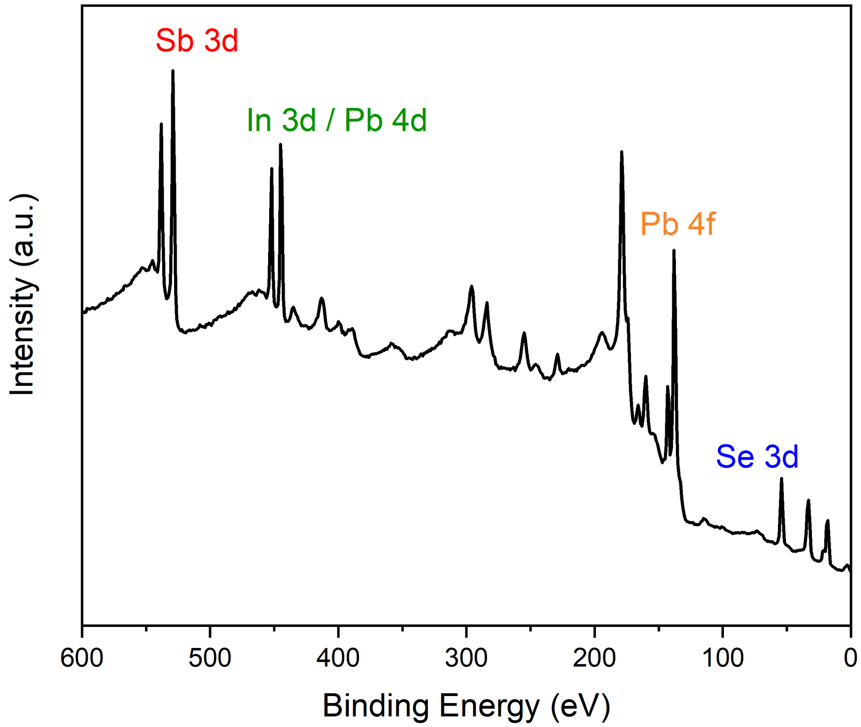

Figure S4. X-ray photoelectron spectra of $Pb_2In_5Sb_9Se_{23}$.

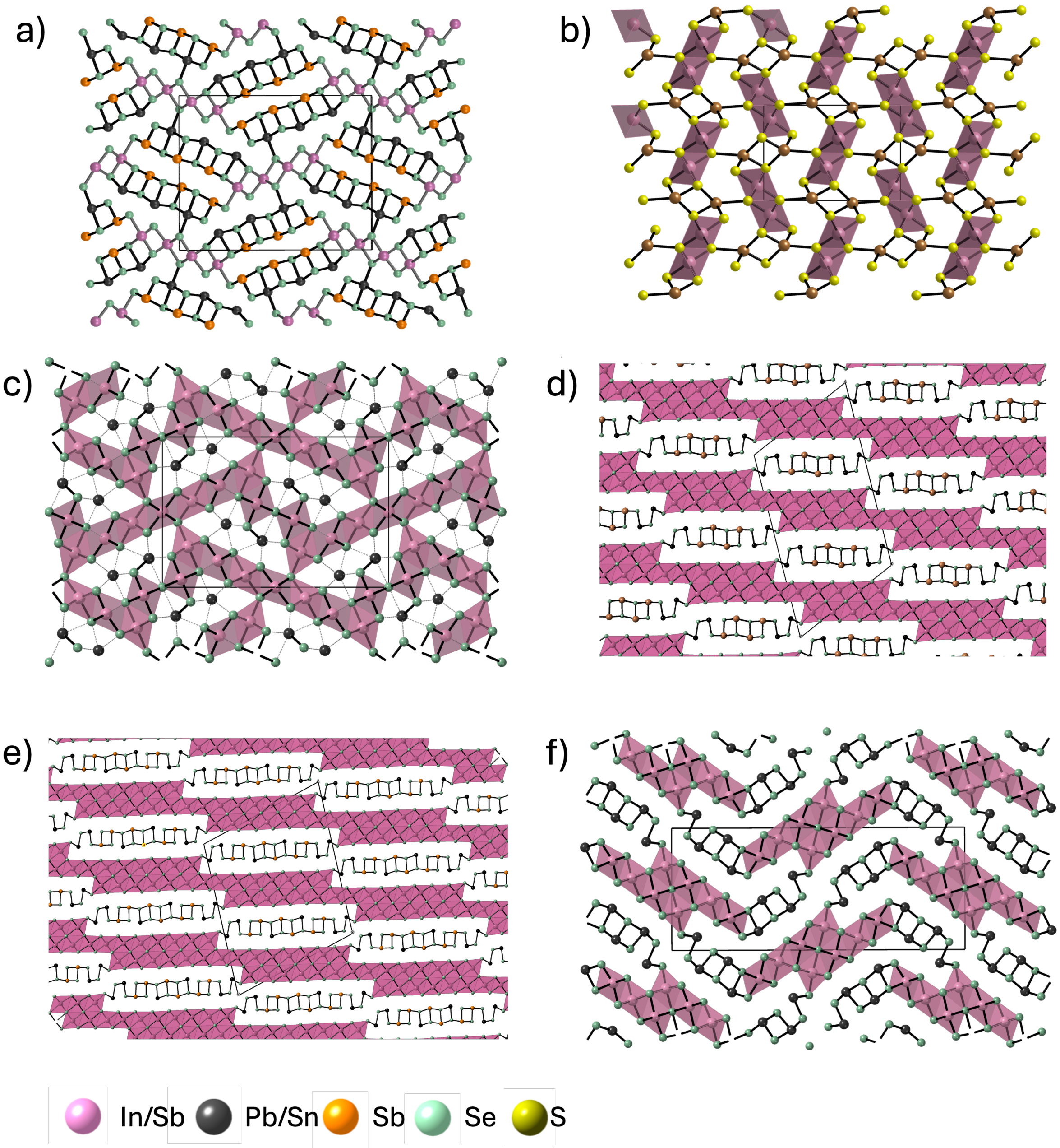

Figure S5. Structural comparison of representative multinary chalcogenides. a) $Pb_4Sb_4Se_{10}$[19], b) $InSbS_3$[20], c) $Pb_5In_8Se_{17}$[21, 22], d) $Sn_4In_5Sb_9Se_{25}$[23], and e) $Sn_{6.13}Pb_{1.87}In_{5.00}Sb_{10.12}Bi_{2.88}Se_{35}$[23], and (f) $M_2In_5Sb_9Se_{23}$ (This work).

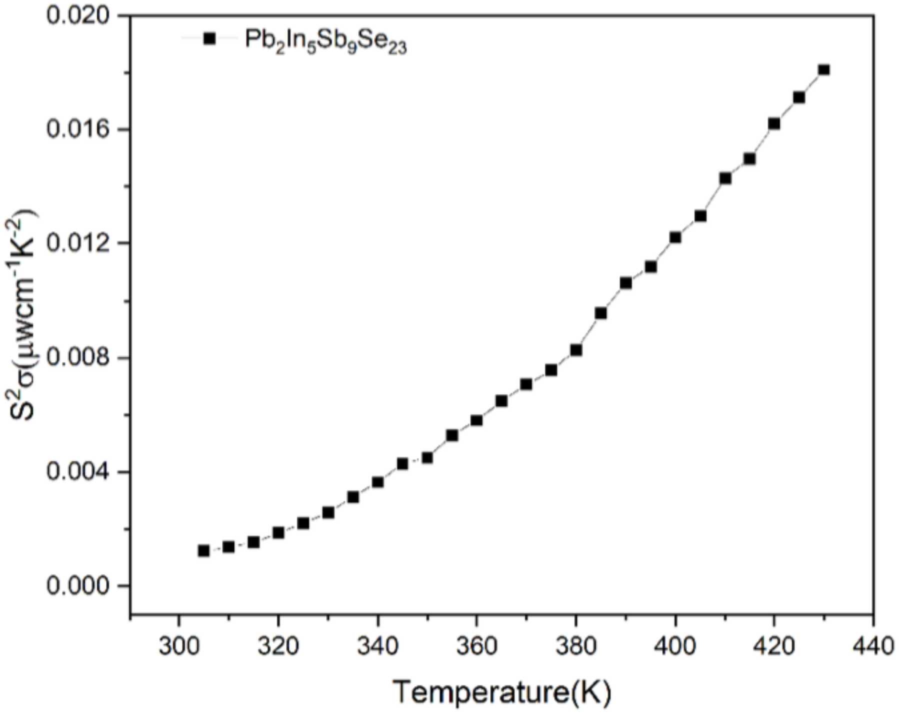

Figure S6. Temperature dependence of the power factor ($S^2\sigma$) for $Pb_2In_5Sb_9Se_{23}$.

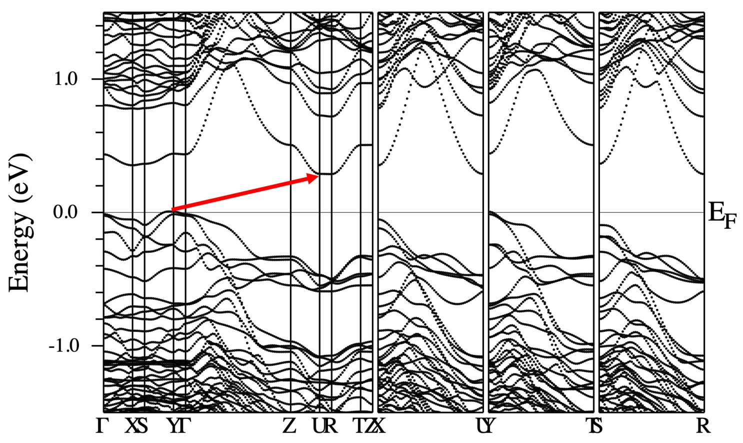

a) Band Structure

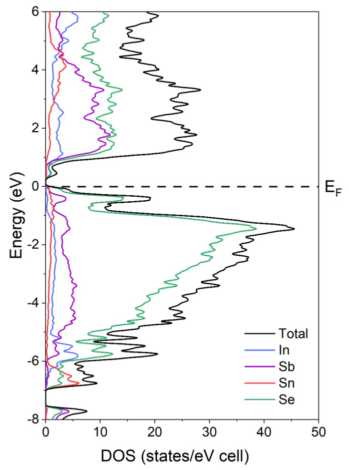

b) DOS and PDOS

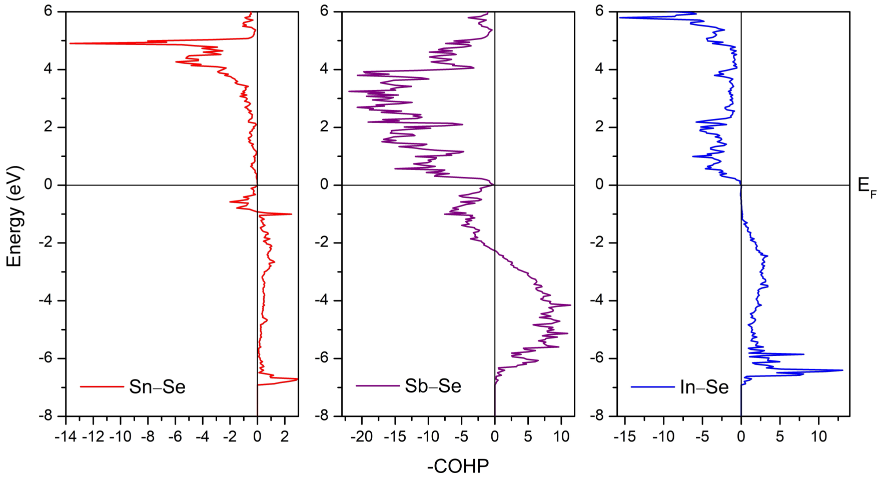

c) −COHP

Figure S7. Electronic-structure results for $Sn_2In_5Sb_9Se_{23}$: (a) band structure showing an indirect band gap, (b) total and partial density of states, and (c) crystal orbital Hamilton population (COHP). The dashed line indicates the Fermi energy (E_f).

Table S1. Atomic coordinates, site occupancies, and isotropic atomic displacement parameters for $M_2In_5Sb_9Se_{23}$ (M = Sn, Pb).

a) $Sn_2In_5Sb_9Se_{23}$

Atom	W.P.	x/a	y/b	z/c	Ueq[a]	Site occ.	
M1	4f	0.81008(11)	0.02064(6)	0	0.0193(4)	In/Sb	0.62/0.38
M2	4e	0.04249(12)	0.05844(5)	0.5	0.0183(4)	In/Sb	0.62/0.38
M3	4f	0.90648(12)	0.13570(6)	0	0.0248(5)	In/Sb	0.62/0.38
M4	4e	0.76344(13)	0.20134(6)	0.5	0.0260(5)	In/Sb	0.62/0.38
M5	4f	0.66009(15)	0.29394(8)	0	0.0388(6)	Sn/Sb	0.45/0.55
M6	4e	0.91193(13)	0.30929(6)	0.5	0.0220(5)	Sn/Sb	0.10/0.90
M7	4f	0.34942(14)	0.09406(6)	0	0.0304(5)	Sn/Sb	0.16/0.84
M8	4e	0.60651(14)	0.08456(7)	0.5	0.0318(5)	Sn/Sb	0.35/0.65
Se1	2a	0	0	0	0.0211(8)	Se	1
Se2	4e	0.8519(2)	0.07726(9)	0.5	0.0292(7)	Se	1
Se3	4f	0.72660(18)	0.15401(9)	0	0.0230(6)	Se	1
Se4	4e	0.21406(16)	0.03198(8)	0.5	0.0169(6)	Se	1
Se5	4f	0.07850(17)	0.10729(8)	0	0.0192(6)	Se	1
Se6	4e	0.94266(18)	0.18456(8)	0.5	0.0223(6)	Se	1
Se7	4f	0.61357(17)	0.24195(8)	0.5	0.0228(6)	Se	1
Se8	4e	0.82364(17)	0.26122(8)	0	0.0210(6)	Se	1
Se9	4f	0.76476(17)	0.34950(8)	0.5	0.0208(6)	Se	1
Se10	4e	0.98767(17)	0.35831(8)	0	0.0209(6)	Se	1
Se11	4f	0.43887(16)	0.05473(8)	0.5	0.0180(6)	Se	1
Se12	4e	0.62893(17)	0.03321(8)	0	0.0193(6)	Se	1

b) $Pb_2In_5Sb_9Se_{23}$

Atom	W.P.	x/a	y/b	z/c	Ueq[a]	Site occ.	
M1	4e	0.68919(7)	0.97931(3)	0.5	0.0172(3)	In/Sb	0.62/0.38
M2	4f	0.54178(7)	0.05838(3)	0	0.0168(3)	In/Sb	0.62/0.38
M3	4e	0.40668(8)	0.13572(3)	0.5	0.0232(3)	In/Sb	0.62/0.38
M4	4f	0.26301(8)	0.20105(3)	0	0.0243(3)	In/Sb	0.62/0.38
M5	4e	0.15428(8)	0.29550(4)	0.5	0.0492(5)	Pb/Sb	0.45/0.55
M6	4f	0.41047(8)	0.30891(3)	0	0.0265(5)	Pb/Sb	0.10/0.90
M7	4e	0.84533(9)	0.09355(3)	0.5	0.0378(5)	Pb/Sb	0.16/0.84
M8	4f	0.89007(8)	0.91376(3)	0	0.0422(5)	Pb/Sb	0.35/0.65
Se1	2a	0.5	0	0.5	0.0207(5)	Se	1
Se2	4f	0.35222(12)	0.07636(5)	0	0.0278(4)	Se	1
Se3	4e	0.22754(11)	0.15366(5)	0.5	0.0212(4)	Se	1
Se4	4f	0.71287(10)	0.03186(4)	0	0.0153(3)	Se	1
Se5	4e	0.57691(11)	0.10692(4)	0.5	0.0175(4)	Se	1
Se6	4f	0.44225(11)	0.18447(4)	0	0.0207(4)	Se	1
Se7	4f	0.11168(11)	0.24079(5)	0	0.0237(4)	Se	1
Se8	4e	0.32221(11)	0.26084(4)	0.5	0.0202(4)	Se	1
Se9	4f	0.26247(11)	0.34946(5)	0	0.0202(4)	Se	1
Se10	4e	0.01418(10)	0.85791(4)	0.5	0.0200(4)	Se	1
Se11	4f	0.06179(10)	0.94542(4)	0	0.0181(4)	Se	1
Se12	4e	0.87056(10)	0.96714(4)	0.5	0.0188(4)	Se	1

Table S2. Interatomic Distances (Å) for $M_2In_5Sb_9Se_{23}$ (M = Sn, Pb).
$M_2In_5Sb_9Se_{23}$

$Sn_2In_5Sb_9Se_{23}$			$Pb_2In_5Sb_9Se_{23}$		
	**Contacts**	**Distance(Å)**		**Contacts**	**Distance(Å)**
M1	Se3	2.6681(3)	M1	Se12	2.6810(19)
M1	Se1×2	2.6808(2)	M1	Se4×2	2.7461(13)
M1	Se10×2	2.9246(2)	M1	Se1	2.8546(11)
M2	Se12×2	2.6813(2)	M1	Se2×2	2.8640(15)
M2	Se1	2.6988(3)	M2	Se4	2.6642(18)
M2	Se5	2.7004(4)	M2	Se5×2	2.6808(12)
M2	Se9×2	2.9672(3)	M2	Se2	2.837(2)
M3	Se2	2.6788(3)	M2	Se1×2	2.9305(8)
M3	Se3×2	2.7393(3)	M3	Se5	2.6814(19)
M3	Se10	2.8626(2)	M3	Se6×2	2.6869(13)
M3	Se9×2	2.8781(3)	M3	Se3	2.689(2)
M4	Se6	2.6580(3)	M3	Se2×2	2.9980(15)
M4	Se2×2	2.7063(3)	M4	Se7	2.611(2)
M5	Se8	2.6070(3)	M4	Se3×2	2.6565(14)
M5	Se5×2	2.6498(3)	M4	Se6	2.680(2)
M5	Se12	2.6793(35)	M4	Se8×2	3.0274(15)
M5	Se4×2	3.0247(27)	M5	Se8	2.735(2)
M6	Se7	2.5662(4)	M5	Se7×2	2.845(2)
M6	Se11×2	2.8551(3)	M6	Se9	2.5854(19)
M6	Se4×2	2.9115(3)	M6	Se10×2	2.8617(14)
M7	Se4	2.6456(4)	M6	Se8×2	2.9202(14)
M7	Se8×2	2.7852(3)	M7	Se10	2.6640(19)
M7	Se7×2	3.1780(3)	M7	Se11×2	2.7808(14)
M7	Se8	2.6070(3)	M7	Se9×2	3.0794(15)
M8	Se5×2	2.6498(3)	M8	Se11	2.740(2)
M8	Se12	2.6793(4)	M8	Se12×2	2.7588(14)
M8	Se4×2	3.0247(3)			

Table S3. Elemental analysis of $Pb_2In_5Sb_9Se_{23}$ from SEM-EDS and ICP.

**$Pb_2In_5Sb_9Se_{23}$**			
	**EDS. atomic%**	**ICP atomic%**	**Ideal atomic%**
**Pb**	**5.3**	**5.3**	**5.1**
**In**	**12.4**	**13.1**	**12.8**
**Sb**	**21.1**	**21.5**	**23.1**
**Se**	**61.2**	**60.7**	**59.0**

Table S4. Screening of synthesis conditions for the Sn analogue using the starting composition $Sn_2In_xSb_{14-x}Se_{23}$ (x = 5, 6, 7)

Entry	Reaction conditions	PXRD result (phase assemblage)
1	RT → 450 °C (48 h), furnace-cooled	$Sn_2In_5Sb_9Se_{23}$ (major) + In_2Se_3 (minor) + unidentified peaks
2	RT → 500 °C (48 h), furnace-cooled	$Sn_2In_5Sb_9Se_{23}$ (major) + In_2Se_3 (minor) + unidentified peaks
3	RT → 550 °C (48 h), furnace-cooled	$Sn_2In_5Sb_9Se_{23}$ (major) + In_2Se_3 (minor) + unidentified peaks
4	RT → 600 °C (48 h), furnace-cooled	$Sn_2In_5Sb_9Se_{23}$ (major) + In_2Se_3 (minor) + unidentified peaks
5	RT → 500 °C (48 h), ice-water quench	$Sn_2In_5Sb_9Se_{23}$ (major) + In_2Se_3 (minor) + unidentified peaks; poorer crystallinity
6	RT → 550 °C (48 h), ice-water quench	$Sn_2In_5Sb_9Se_{23}$ (major) + In_2Se_3 (minor) + unidentified peaks; poorer crystallinity
7	RT → 600 °C (48 h), ice-water quench	$Sn_2In_5Sb_9Se_{23}$ (major) + In_2Se_3 (minor) + unidentified peaks; poorer crystallinity
8	RT → 500 °C (48 h), slow cooling	$Sn_2In_5Sb_9Se_{23}$ (major) + In_2Se_3 (minor) + unidentified peaks
9	RT → 550 °C (48 h), slow cooling	$Sn_2In_5Sb_9Se_{23}$ (major) + In_2Se_3 (minor) + unidentified peaks
10	RT → 600 °C (48 h), slow cooling	$Sn_2In_5Sb_9Se_{23}$ (major) + In_2Se_3 (minor) + unidentified peaks

Notes: “Major/minor” refers to relative intensities in the PXRD patterns. “Unidentified peaks” indicate additional weak reflections not attributable to the phases listed above.

Table S5. Bond-valance sum for $M_2In_5Sb_9Se_{23}$ (M = Sn, Pb).[24, 25]

Site	Element	Occupancy	$Sn_2In_5Sb_9Se_{23}$	$Pb_2In_5Sb_9Se_{23}$
M1	Sb	0.38	3.33	3.35
	In	0.62	2.54	2.56
M2	Sb	0.38	3.48	3.50
	In	0.62	2.65	2.67
M3	Sb	0.38	3.57	3.55
	In	0.62	2.73	2.71
M4	Sb	0.38	3.85	3.80
	In	0.62	2.94	2.90
M5	Sb	0.55	2.32	1.98
	Sn/Pb	0.45	2.04	2.67
M6	Sb	0.91	2.73	2.65
	Sn/Pb	0.09	2.88	3.60
M7	Sb	0.84	2.62	2.41
	Sn/Pb	0.15	2.22	3.36
M8	Sb	0.65	2.44	2.08
	Sn/Pb	0.35	2.29	3.01
Se1	Se	1.00	1.87	2.08
Se2	Se	1.00	2.08	1.75
Se3	Se	1.00	1.97	2.04
Se4	Se	1.00	1.90	1.75
Se5	Se	1.00	2.05	1.93
Se6	Se	1.00	1.73	1.88
Se7	Se	1.00	2.13	1.86
Se8	Se	1.00	1.98	2.02
Se9	Se	1.00	2.03	1.96
Se10	Se	1.00	1.70	2.03
Se11	Se	1.00	1.91	1.89
Se12	Se	1.00	1.72	1.96

References

(1) *APEX2 v2.1*; Bruker AXS Inc., Madison, Wisconsin, USA: 2006.
(2) Blessing, R. H. An empirical correction for absorption anisotropy. *Acta Crystallogr A* **1995**, *51 (Pt 1)* (1), 33–38. DOI: 10.1107/s0108767394005726 From NLM Medline.
(3) Shyni, P.; Pradyumnan, P. P. Fermi level tuning in modified Bi2Te3 system for thermoelectric applications. *RSC Adv* **2021**, *11* (8), 4539–4546. DOI: 10.1039/d0ra09278a From NLM PubMed-not-MEDLINE.
(4) Zhou, C. J.; Lee, Y. K.; Yu, Y.; Byun, S.; Luo, Z. Z.; Lee, H.; Ge, B. Z.; Lee, Y. L.; Chen, X. Q.; Lee, J. Y.; et al. Polycrystalline SnSe with a thermoelectric figure of merit greater than the single crystal. *Nat. Mater.* **2021**, *20* (10), 1378–+. DOI: 10.1038/s41563-021-01064-6.
(5) Su, B.; Jiang, Y.; Zhuang, H. L.; Han, Z.; Yu, J.; Hu, H.; Li, J. W.; Li, H.; He, Y. X.; Chen, L.; et al. Ultralow Lattice Thermal Conductivity and High ZT of n-Type Polycrystalline SnSe Realized by Liquid Phase Sintering. *Research* **2025**, *8*, 0962. DOI: 10.34133/research.0962 From NLM PubMed-not-MEDLINE.
(6) Kim, H. S.; Gibbs, Z. M.; Tang, Y. L.; Wang, H.; Snyder, G. J. Characterization of Lorenz number with Seebeck coefficient measurement. *Apl Materials* **2015**, *3* (4), 041506. DOI: 10.1063/1.4908244.
(7) Ioffe, A. F. *Semiconductor Thermoelements And Thermoelectric Cooling*; Infosearch, 1957.
(8) Blaha, P.; Schwarz, K.; Tran, F.; Laskowski, R.; Madsen, G. K. H.; Marks, L. D. WIEN2k: An APW+lo program for calculating the properties of solids. *J. Chem. Phys.* **2020**, *152* (7), 074101. DOI: 10.1063/1.5143061 From NLM PubMed-not-MEDLINE.
(9) Schwarz, K.; Blaha, P.; Madsen, G. K. H. Electronic structure calculations of solids using the WIEN2k package for material sciences. *Comput. Phys. Commun.* **2002**, *147* (1-2), 71–76. DOI: Doi 10.1016/S0010-4655(02)00206-0.
(10) Madsen, G. K. H.; Blaha, P.; Schwarz, K.; Sjöstedt, E.; Nordström, L. Efficient linearization of the augmented plane-wave method. *Phys. Rev. B* **2001**, *64* (19), 195134. DOI: 10.1103/PhysRevB.64.195134.
(11) Perdew, J. P.; Burke, K.; Ernzerhof, M. Generalized Gradient Approximation Made Simple. *Phys. Rev. Lett.* **1996**, *77* (18), 3865–3868. DOI: 10.1103/PhysRevLett.77.3865 From NLM Publisher.
(12) Koller, D.; Tran, F.; Blaha, P. Merits and limits of the modified Becke-Johnson exchange potential. *Phys. Rev. B* **2011**, *83* (19), 195134. DOI: ARTN 195134
10.1103/PhysRevB.83.195134.
(13) Blochl, P. E.; Jepsen, O.; Andersen, O. K. Improved tetrahedron method for Brillouin-zone integrations. *Phys Rev B Condens Matter* **1994**, *49* (23), 16223–16233. DOI: 10.1103/physrevb.49.16223 From NLM PubMed-not-MEDLINE.
(14) Setyawan, W.; Curtarolo, S. High-throughput electronic band structure calculations: Challenges and tools. *Comput. Mater. Sci.* **2010**, *49* (2), 299–312. DOI: 10.1016/j.commatsci.2010.05.010.
(15) Koelling, D. D.; Harmon, B. N. A technique for relativistic spin-polarised calculations. *J. Phys. C* **1977**, *10* (16), 3107. DOI: 10.1088/0022-3719/10/16/019.

(16) Dronskowski, R.; Blochl, P. E. Crystal Orbital Hamilton Populations (Cohp) - Energy-Resolved Visualization of Chemical Bonding in Solids Based on Density-Functional Calculations. *J. Phys. Chem.* **1993**, *97* (33), 8617–8624. DOI: DOI 10.1021/j100135a014.
(17) *The STUTTGART TB-LMTO-ASA program*; Max-Planck-Institut für Festkörperforschung: Stuttgart, Germany, 2000.
(18) Skriver, H. L. *The LMTO Method*.
(19) Skowron, A.; Brown, I. D. Structure of Antimony Lead Selenide, Pb4Sb4Se10, a Selenium Analogue of Cosalite *Acta Crystallogr. C.* **1990**, *46* (12), 2287–2291. DOI: Doi 10.1107/S010827019000405x.
(20) Paulus, H.; Fuess, H. Crystal structure of indium antimony trisulflde, InSbS3. *Z. Kristallogr.* **1992**, *198* (1-4), 125–126. DOI: doi:10.1524/zkri.1992.198.14.125 (accessed 2025-10-12).
(21) Winkler, H. P.; Topa, D.; Keller, E. Pb5In3Bi5S17 [Pb4.94(3)In3.05(3)Bi4.99(3)S17] and its structural relationship with Pb4In3Bi7S18. *Acta Crystallogr. C.* **2012**, *68* (7), I45–I49. DOI: 10.1107/S0108270112021865.
(22) Ginderow, D. Structures cristallines de Pb4In9S17 et Pb3In6,67S13. *Acta Crystallogr. B.* **1978**, *34* (6), 1804–1811. DOI: 10.1107/s0567740878006731.
(23) Chen, G. R.; Wang, M. F.; Lee, C. S. Synthesis and characterization of new multinary selenides Sn4In5Sb9Se25 and Sn6.13Pb1.87In5.00Sb10.12Bi2.88Se35. *J. Solid State Chem.* **2022**, *307*, 122855. DOI: 10.1016/j.jssc.2021.122855.
(24) Brese, N. E.; Okeeffe, M. Bond-Valence Parameters for Solids. *Acta Crystallogr. B.* **1991**, *47* (2), 192–197. DOI: Doi 10.1107/S0108768190011041.
(25) Brown, I. D. Recent developments in the methods and applications of the bond valence model. *Chem. Rev.* **2009**, *109* (12), 6858–6919. DOI: 10.1021/cr900053k.
